# Medical Prizes

**Published:** 1859-09

**Authors:** 


MEDICAL PRIZES.—The subjects for the Jacksonian Prizes, as announced for the year 1860 by the Council of the College of Surgeons, are, "The Healthy and Morbid Anatomy of the Prostate Gland," and "A Description of the Diseased Conditions of the Knee-joint which require Amputation of the Limb, and of those Conditions which are Favorable for Excision of the Joint, with an Explanation of the Relative Advantages of both Operations, so far as can be ascertained by Cases properly authenticated."

M. Lombard, President of the Medical Society of Geneva, has announced that the Society will award, in 1860, a prize of two hundred dollars, and a second prize of one hundred dollars, to the authors of the two best papers on smallpox, varioloid, varicella, vaccinia, and re-vaccinations.

A prize of sixty dollars is offered by the Imperial Society of Medicine of Bordeaux, for the best essay on the "Prophylaxis of Consumption."

For the best memoir on the "Value of Caustics in the Treatment of Cancer," the Medical Society of Toulouse offers a prize of sixty dollars; the prize for 1861 will be for an essay on "The Influence of Cultivation upon Plants employed in Medicine."

The Medico-Chirurgical Academy of Ferrara will award a prize of two hundred Roman crowns, in 1862, for the best essay on "Mental Diseases in their Relation to Legal Medicine."

The subject for the prize of one hundred Roman crowns, offered by the Medico-Chirurgical Society of Bologna, is "Suckling, Considered in Relation to the Diseases of the Nurse and the Infant."

The Trustees of the Fiske Fund announce for 1860 two prizes, of one hundred dollars each, for the best papers on the following subjects: "Diphtheria; its Nature and Treatment, with an Account of the History of its Prevalence in Different Countries;" and "The Morbid Effects of Retention in the Blood of the Elements of the Urinary Secretion."

